# Advances in Perinatal Depression: A Focus on Screening and Treatment

**DOI:** 10.3390/jcm15124465

**Published:** 2026-06-09

**Authors:** Haonan Shui, Xiaotong Cao, Xuemei Zhang

**Affiliations:** Department of Obstetrics, The First Affiliated Hospital of Chongqing Medical University, Chongqing 400016, China; shn18048697276@163.com (H.S.); xiaotong212125@163.com (X.C.)

**Keywords:** perinatal depression, screening, treatment, precision medicine, collaborative care models

## Abstract

Perinatal depression (PND), encompassing both major and minor depressive episodes during pregnancy and up to one year postpartum, is a prevalent and debilitating condition with profound consequences for maternal, infant, and family well-being. Clinical screening of PND remains challenging due to obstacles in early detection, symptom overlap with normal perinatal experiences, lack of standardized screening protocols, and considerable interpatient variability. Furthermore, the complexity of treating PND arises from multiple interconnected factors, including medication safety considerations during pregnancy and lactation, information barriers resulting from the separation of mental health services from obstetric and pediatric care systems, and unique sociocultural obstacles. The absence of systematically integrated care pathways often leads to severe and potentially irreversible outcomes for both mothers and infants. Hence, this review summarizes recent advances in PND screening and treatment, emphasizing the critical transition toward integrated or collaborative care models. Notably, future efforts should focus on overcoming implementation barriers, digital health solutions, task-sharing frameworks, and personalized treatment strategies to ensure equitable access to these innovations for affected populations.

## 1. Introduction

PND encompasses major or minor depressive episodes occurring during pregnancy (antenatal depression) or within the first year postpartum (postpartum depression). It is one of the most common complications of the perinatal period, imposing a substantial burden on maternal health and serving as a critical determinant of infant cognitive, emotional, and behavioral development. In this review, “PND” refers to unipolar depressive disorders without psychotic features, excluding severe postpartum psychiatric emergencies such as psychosis or acute suicidality, which require immediate specialized care [1,2]. Furthermore, the detrimental effects of PND extend beyond the mother-infant dyad, impacting family system stability and incurring considerable societal healthcare costs [3,4,5,6]. However, in stark contrast to this severe disease burden, the current clinical systems for PND diagnosis and treatment remain inadequate, resulting in a significant service gap. Therefore, the systematic improvement of PND screening and treatment strategies has become an urgent priority.

The diagnosis of PND presents inherent clinical challenges. A primary diagnostic difficulty lies in the significant symptom overlap between core manifestations of PND, including fatigue, sleep disturbances, and changes in appetite, and the normal physiological experiences of pregnancy and the postpartum period. This overlap often complicates early identification [7,8,9,10]. Further compounding the issue are psychosocial barriers, including stigma, fear of judgment, and limited mental health awareness, which frequently lead to symptom non-disclosure and missed opportunities for intervention. Moreover, structural obstacles within healthcare systems exacerbate these challenges. The conventional siloing of medical specialties often results in the separation of mental health services from routine obstetric and pediatric care, creating significant barriers to timely, integrated, and accessible treatment for affected individuals [11,12].

In recent years, however, the landscape of PND care has begun to evolve, driven by growing recognition of its importance. Significant advances have been made in two key areas: screening and treatment. There is a strengthened consensus advocating for universal, routine screening using validated tools like the Edinburgh Postnatal Depression Scale (EPDS) across the entire perinatal timeline [13,14,15,16]. This screening recommendation is consistent with ACOG Clinical Practice Guideline No. 4 (screening and diagnosis of perinatal mental health conditions) [17] and the treatment discussion aligns with ACOG Clinical Practice Guideline No. 5 (treatment and management with a focus on psychopharmacotherapy) [18] and NICE CG192 (recognition, assessment, treatment, and service organization) [19]. Concurrently, the therapeutic strategy has expanded beyond traditional models. Evidence-based psychotherapies, such as interpersonal therapy (IPT) [20,21] and cognitive-behavioral therapy (CBT) [22,23], have been specifically adapted for perinatal populations. The evidence base for the safe use of pharmacotherapy, particularly selective serotonin reuptake inhibitors (SSRIs), during pregnancy and lactation continues to grow, facilitating more informed risk-benefit discussions [24,25,26].

This narrative review summarizes recent evidence on the screening and treatment of PND, with particular attention to implementation barriers, integrated care models, and the translational status of biomarkers and digital tools. The novelty of this review lies in its integration of screening, digital monitoring, biomarkers, and collaborative care within a single clinical framework—distinguishing it from existing guidelines or systematic reviews that focus on isolated aspects of PND care. Overall, the most promising advancement in the field is the paradigm shift toward integrated or collaborative care models [27,28,29], which seek to bridge the critical gap between screening and treatment by embedding mental health services within routine maternity care. This review aims to synthesize recent progress in PND screening and treatment, expound newly proposed assessment tools and systematic interventions, and evaluate the efficacy of comprehensive care frameworks. Finally, it will identify persistent challenges and outline future directions for research and practice, with the ultimate goal of advancing systematic, accessible, and patient-centered approaches to PND care.

## 2. Methods

This is a narrative review of the literature on PND, focusing on screening and treatment. A literature search was conducted in PubMed, Scopus, and Web of Science for articles published between 2015 and 2025. The search strategy combined terms related to perinatal depression (“perinatal depression,” “postpartum depression,” “antenatal depression”) with terms related to the main topics of this review (“screening,” “EPDS,” “biomarkers,” “digital health,” “collaborative care,” “psychotherapy,” “antidepressants,” “brexanolone,” “zuranolone,” and “neuromodulation”). Only peer-reviewed articles, systematic reviews, meta-analyses, randomized controlled trials, and clinical guidelines published in English were included. Reference lists of retrieved articles were manually screened for additional relevant studies. Because this is a narrative review, SANRA (Scale for the Assessment of Narrative Review Articles) guidelines were used to ensure quality and transparency. No quantitative synthesis or meta-analysis was performed.

## 3. Pathophysiological Mechanisms

### 3.1. Neuroendocrine Hypothesis

**Figure 1:**
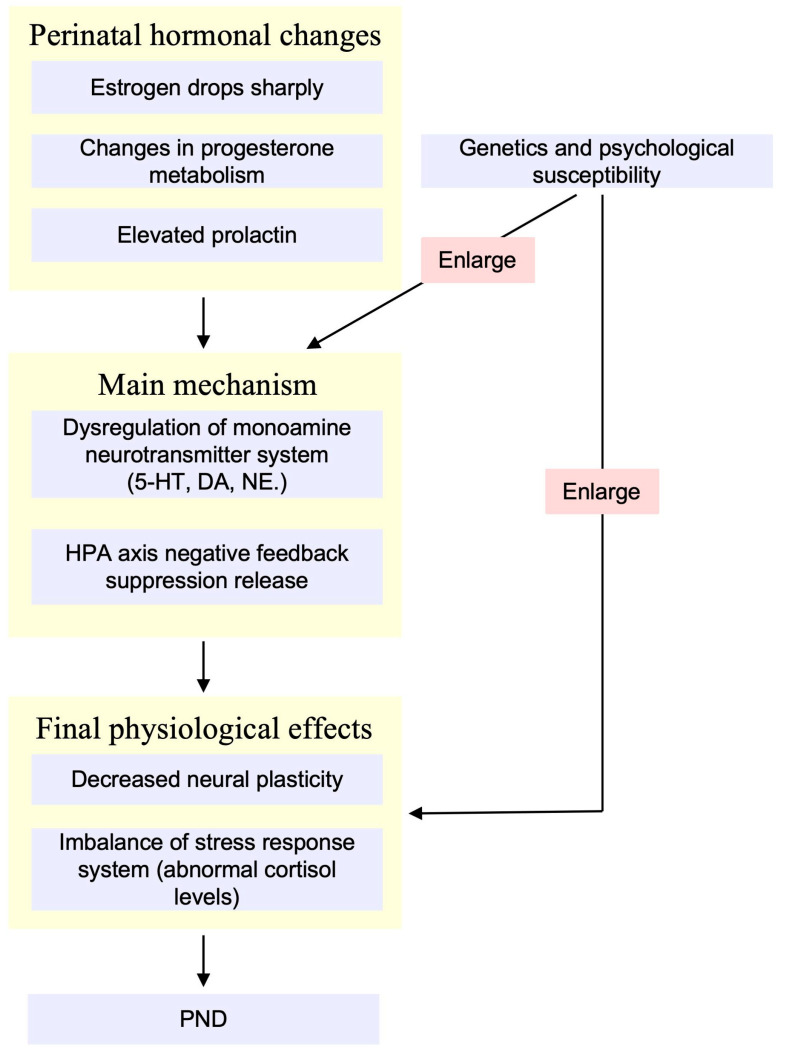
Neuroendocrine hypothesis explains the pathogenesis of PND. 5-HT: serotonin; DA: dopamine; NE: norepinephrine; HPA: hypothalamic-pituitary-adrenal axis; PND: perinatal depression.

The neuroendocrine hypothesis represents one of the central theoretical frameworks for explaining the pathogenesis of PND. This model proposes that the sharp fluctuations in reproductive hormones, such as estrogen (E1), progesterone (P4), and prolactin (PRL) during the perinatal period do not directly cause depression. Instead, they act as a physiological trigger that disrupts the balance of key neurotransmitter systems in the brain and dysregulates the stress-response function of the hypothalamic-pituitary-adrenal (HPA) axis, thereby precipitating depressive symptoms in individuals with genetic or psychological vulnerability [30,31,32,33,34]. The core pathway of this hypothesis can be summarized by the cascade of events illustrated in Figure 1.

#### 3.1.1. Interaction of Sex Hormone Fluctuations with Monoamine Neurotransmitter Systems

Monoamine neurotransmitters, such as serotonin (5-HT), dopamine (DA), and norepinephrine (NE), play a critical role in regulating mood, motivation, and reward. Sex hormones modulate their synthesis, release, and signaling through multiple mechanisms.

Firstly, estrogen exerts extensive regulatory effects on the 5-HT system. It upregulates the expression and function of 5-HT receptors, enhances the expression of tryptophan hydroxylase (TPH), the rate-limiting enzyme in 5-HT synthesis, and inhibits the activity of the serotonin reuptake transporter. Consequently, elevated estrogen levels potentiate serotonergic neurotransmission, thereby promoting mood stability [35,36]. However, following delivery, estrogen levels drop precipitously to baseline within 24–48 h. This sharp decline withdraws essential support for the 5-HT system, leading to a rapid reduction in neurotransmission efficacy that constitutes a key biological basis for postpartum depression [37,38,39].

Then, P4 and its neuroactive metabolite, allopregnanolone (ALLO), act as positive allosteric modulators of the γ-aminobutyric acid type A (GABAA) receptor, conferring anxiolytic and sedative effects. The rapid postpartum decline in P4 levels reduces ALLO availability, diminishing GABAergic tone and potentially contributing to emotional instability and anxiety [40,41,42,43]. Furthermore, certain intermediate metabolites of P4 such as pregnenolone may act as competitive antagonists at the GABAA receptor, further disrupting neuronal stability [44,45,46].

Besides, PRL levels rise significantly during the perinatal period. As its secretion is tonically inhibited by hypothalamic dopaminergic signaling, elevated PRL may feedback to suppress dopaminergic activity. This inhibition can impair function in the mesolimbic reward pathway, leading to reduced motivation and anhedonia, a core symptom of depression [47,48].

#### 3.1.2. HPA Axis Dysregulation: The Role of Cortisol Dynamics in Depressive Symptoms

The HPA axis, a central component of the body’s stress response system, undergoes significant adaptations during the perinatal period that are closely linked to the development of depression. In patients with PND, the negative feedback mechanism of the HPA axis is frequently impaired. Despite elevated cortisol levels, there is a failure to adequately suppress further activation of the hypothalamus and pituitary, a dysfunction potentially associated with compromised glucocorticoid receptor function in the hippocampus [49]. Affected individuals often exhibit distinct abnormalities in cortisol secretion patterns, including: (1) elevated basal levels: reflected by increased 24-h urinary free cortisol or elevated daytime baseline cortisol; (2) a flattened diurnal rhythm: characterized by an attenuated morning peak and reduced variability throughout the day; and (3) hyperactive stress responsiveness: manifesting as an exaggerated and prolonged cortisol response to psychological or physiological challenges [50,51,52,53,54]. Furthermore, estrogen enhances corticotropin-releasing hormone (CRH) gene expression, whereas P4 and its metabolite ALLO exert inhibitory effects on HPA axis activity. Consequently, the dramatic fluctuations in perinatal hormone levels directly impact HPA axis stability. The postpartum decline in progesterone and allopregnanolone effectively removes this inhibitory influence, potentially leading to HPA axis overactivation.

Overall, the neuroendocrine hypothesis [55,56,57] posits that perinatal depression arises from a vicious cycle involving sex hormone fluctuations, monoaminergic neurotransmitter systems, and HPA axis dysfunction. In genetically susceptible individuals, perinatal hormonal changes act as a physiological trigger, impairing serotonergic and dopaminergic function and reducing inhibitory control over the HPA axis. Subsequent HPA axis overactivation elevates cortisol levels, which further suppresses 5-HT synthesis and hippocampal neuroplasticity, exacerbating emotional dysregulation. Thus, PND reflects an interaction between physiological hormonal changes and neurobiological vulnerability, disrupting neurotransmitter and stress response systems. This mechanism underpins targeted therapeutic strategies focusing on HPA axis modulation or neuroactive steroids such as allopregnanolone analogs.

### 3.2. Neuroinflammation and Immune Activation

**Figure 2:**
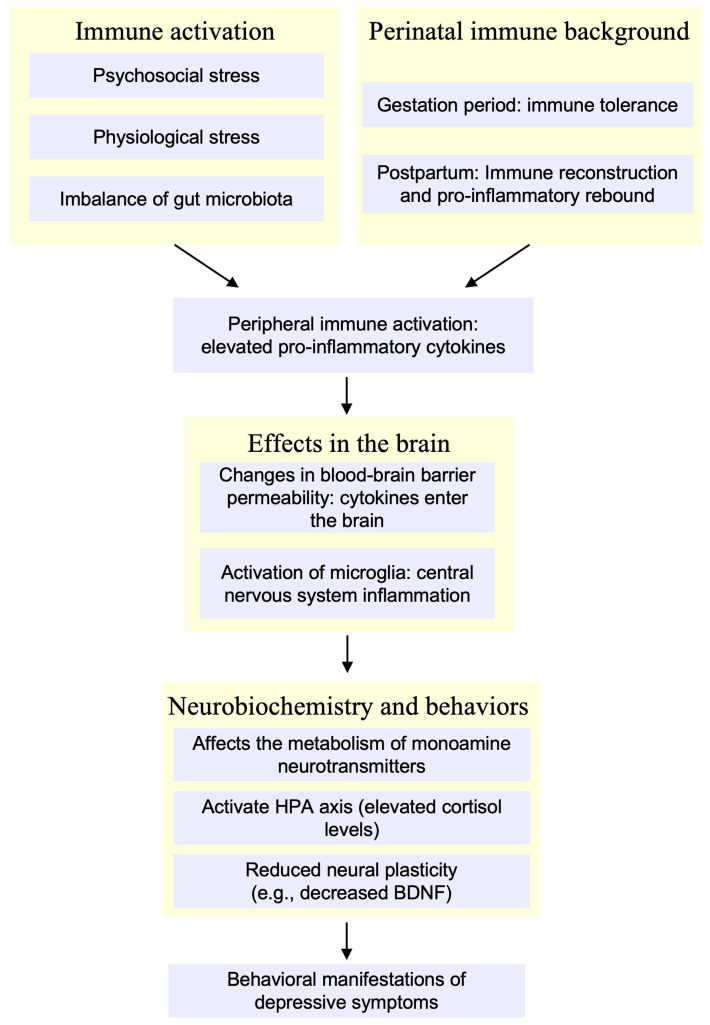
Neuroinflammation and immune activation explain the pathogenesis of perinatal depression. BDNF: brain-derived neurotrophic factor.

The neuroinflammation and immune activation hypothesis offers a critical perspective for understanding PND. It proposes that adaptive immunological changes inherent to the perinatal period may become disordered under specific conditions. Such dysregulation promotes a pro-inflammatory state within the central nervous system, ultimately contributing to the development of depressive symptoms. The core pathway involves intricate crosstalk between peripheral immunity and the brain, with the principal mechanisms summarized in the cascade model presented in Figure 2 [58].

#### 3.2.1. Balancing Immune Tolerance with Pro-Inflammatory Cytokine Activity in Pregnancy

A successful pregnancy is contingent upon the establishment of maternal immune tolerance to prevent rejection of the semi-allogeneic fetus. This adaptive state is characterized by a shift toward Th2-type cytokine dominance, including elevated IL-4 and IL-10, enhanced regulatory T-cell activity, and concomitant suppression of pro-inflammatory Th1 responses such as IL-2 and IFN-γ [59,60,61]. However, this immunomodulation presents a physiological paradox. As term approaches, a controlled pro-inflammatory shift becomes necessary to initiate parturition. This transition from an anti-inflammatory Th2 environment to a pro-inflammatory Th1-dominated environment serves as a critical signal for labor, facilitating cervical remodeling and uterine contractions. Key mediators include cytokines such as IL-6, TNF-α, and IL-1β, whose levels rise physiologically during this process. Following delivery, the expulsion of the placenta, which serves as a pivotal immunomodulatory organ, together with the rapid decline in P4 and other immunoregulatory hormones, may trigger a rebound immune activation. This may result in an excessive pro-inflammatory state [62,63,64,65,66]. In immunologically vulnerable individuals, such as those with underlying chronic inflammation or predisposing genetic variants, this normative inflammatory transition may become dysregulated. The resulting systemic low-grade inflammation enables elevated pro-inflammatory cytokines, including IL-6 and TNF-α, to either cross the blood-brain barrier or initiate signaling through cerebral endothelial cells, ultimately contribute to neuroinflammation and the pathophysiology of perinatal depression [67].

#### 3.2.2. The Microbiota–Gut–Brain Axis: Modulating Mood Through Perinatal Gut Microbiota Changes

The perinatal period is characterized by significant fluctuations in gut microbiota composition, which profoundly influence central nervous system function through the microbiota–gut–brain axis [68,69,70,71]. Various factors during late pregnancy and the postpartum period, such as hormonal changes, dietary modifications, mode of delivery, antibiotic exposure, and psychological stress, can lead to microbial dysbiosis. This imbalance compromises intestinal barrier integrity, facilitating the translocation of bacterial metabolites including lipopolysaccharide into systemic circulation. These compounds may trigger a systemic inflammatory response, enabling pro-inflammatory cytokines to communicate with the brain [72,73,74]. Additionally, gut microbiota also directly regulate synthesis of neuroactive compounds (such as GABA, serotonin, dopamine), with dysbiosis disrupting their homeostasis [75,76]. Furthermore, gut microbes calibrate the stress response set-point of the HPA axis, influencing cortisol secretion and increasing depression susceptibility [77].

Overall, the neuroinflammation and immune activation hypothesis provides a mechanistic framework for perinatal depression. Under certain conditions, such as psychological stress and gut microbiota dysbiosis, the physiological immune adaptations of the perinatal period may progress to a pathological inflammatory state. This pathological cascade contributes to the development of PND through several distinct mechanisms, including the disruption of neurotransmitter balance, dysregulation of HPA axis function, and impairment of neural plasticity. These findings provide a compelling scientific basis for developing targeted therapeutic strategies for PND, such as anti-inflammatory treatments and microbiota-based interventions employing probiotics or prebiotics [78].

### 3.3. Genetic and Epigenetic Insights

**Figure 3:**
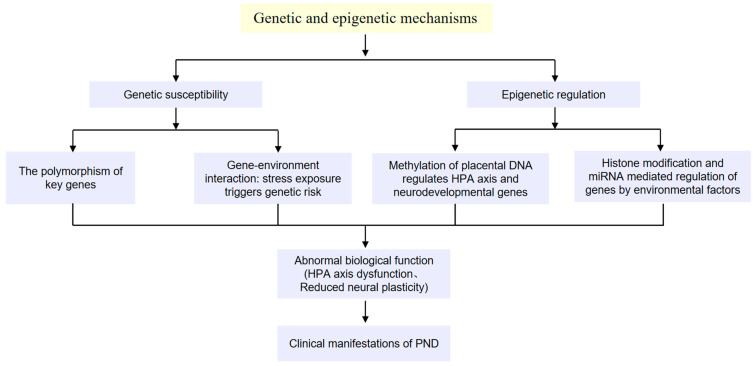
Explanation of the pathogenesis of PND from the perspectives of genetics and epigenetics. 5-HTTLPR: serotonin transporter gene; miRNA: microRNA; NR3C1: glucocorticoid receptor gene; OXTR: oxytocin receptor gene.

The pathogenesis of PND involves a complex interplay between genetic susceptibility and epigenetic regulation. Recent evidence indicates that specific genetic polymorphisms and their interaction with environmental factors, combined with dynamic epigenetic modifications, collectively constitute the molecular basis of PND. The core mechanisms can be systematically illustrated by the framework presented in Figure 3.

#### 3.3.1. Genetic Susceptibility: The Role of Gene-Environment Interactions

Susceptibility genes such as the short allele (S allele) in the promoter region of the serotonin transporter gene (5-HTTLPR) are associated with reduced 5-HTT expression, which impairs synaptic serotonin reuptake and increases the risk of depression under stressful conditions [79,80,81]. Cohort studies have demonstrated that mothers carrying the S allele exhibit a significantly higher incidence of PND when exposed to postpartum stressors. Besides, the Met allele of the brain-derived neurotrophic factor (BDNF) Val66Met polymorphism leads to decreased BDNF secretion, impairing hippocampal neuroplasticity and disrupting the negative feedback regulation of the HPA axis [82,83,84]. Meta-analyses have confirmed a positive correlation between this polymorphism and the severity of postpartum depression. Importantly, the effects of these genetic susceptibilities often require interaction with environmental factors to manifest clinically. For example, carriers of the 5-HTTLPR S allele who experience adverse prenatal life events, such as lack of partner support, show a significantly elevated risk of developing PND compared to non-carriers [85]. Such gene-environment interactions are frequently mediated by epigenetic mechanisms, including DNA methylation, resulting in the biological embedding of stress responses [86].

#### 3.3.2. Epigenetic Modifications: From Dynamic Regulation to Biomarker Potential

Epigenetic modifications play a critical regulatory role in PND through their dynamic changes and hold significant promise as biomarkers. The placenta, acting as a critical maternal-fetal interface, exhibits DNA methylation patterns that sensitively reflect the cumulative effects of prenatal environmental exposures [87,88]. For instance, hypermethylation of the glucocorticoid receptor gene (NR3C1) in the placenta may impaired negative feedback of the HPA axis, and subsequent postpartum cortisol dysregulation [89,90]. Similarly, hypermethylation of the oxytocin receptor gene (OXTR) correlates with impaired mother-infant interactions and postnatal mood disturbances, suggesting its potential as a specific biomarker for PND subtypes with prominent social-behavioral deficits [91,92,93]. Beyond placental markers, easily accessible peripheral blood-based epigenetic signatures, such as differentially expressed miRNAs show considerable translational potential for early PND detection [94,95,96]. These miRNAs are implicated in disease pathogenesis likely through their regulation of inflammatory pathways and genes involved in neuroplasticity.

Overall, genetic susceptibility establishes a baseline level of individual risk for perinatal depression, whereas prenatal environmental exposures, including psychological stress and nutritional status, dynamically modulate gene expression via epigenetic mechanisms. These modifications subsequently contribute to dysregulation of the HPA axis, neurotransmitter imbalances, and impaired neural plasticity. This integrated framework enhances our comprehension of PND heterogeneity while providing novel directions for developing early-warning biomarkers, such as placental methylation patterns and peripheral miRNA profiles, alongside targeted therapeutic strategies including demethylating agents and nutrition-based epigenetic interventions [97]. Most of the evidence discussed in Section 3.1, Section 3.2 and Section 3.3 is correlational or derived from preclinical models. Therefore, causal interpretations should be made with caution. The main pathophysiological mechanisms discussed above are summarized in Table 1.

## 4. Screening and Diagnosis of Perinatal Depression: Integration of Multidimensional Strategies

Early identification and accurate diagnosis of PND are key to improving maternal and infant prognosis. The current practice and research have surpassed single scale screening and are evolving towards a multidimensional model that integrates clinical evaluation, biomarkers, and digital technology.

Screening, diagnosis, and risk prediction serve distinct purposes in perinatal depression care. Screening employs brief instruments (e.g., EPDS, PHQ-9) to identify probable cases, diagnosis requires clinical confirmation by a trained professional, and risk prediction aims to identify individuals at elevated risk before symptom onset. The tools discussed in this section should not be considered interchangeable.

### 4.1. Standardized Scales: Cornerstone, Optimization and Limitations

The EPDS remains the most widely used global tool for screening PND. Its primary advantages lie in its brevity, ease of administration, and high acceptability, making it suitable for primary care settings and routine obstetric follow-ups. However, as its application has expanded across diverse global populations, several limitations have become increasingly apparent, primarily in the following three aspects. Firstly, there are population differences in its sensitivity and specificity [98,99,100]. Multiple systematic reviews indicate that the optimal cutoff scores for the EPDS vary across countries, ethnicities, and cultural contexts. For instance, in some Asian populations, commonly used cutoff values may be too low, resulting in insufficient sensitivity, whereas in certain high-income welfare societies, the same cutoff may exhibit reduced specificity, leading to false-positive results. These variations highlight that the interpretation of EPDS scores requires localized validation based on population-specific characteristics rather than simple reliance on international norms. Secondly, the scale demonstrates limited cross-cultural adaptability [101,102,103,104]. The emotional expressions referenced in certain items (e.g., “self-blame” or “self-accusation”) may carry significantly different connotations and levels of acceptability across cultural contexts. Without rigorous translation, back-translation, and cultural adaptation processes, respondents’ understanding of these items may be biased, compromising the validity of the screening. Therefore, the EPDS must undergo localized reliability and validity testing before application rather than being used via direct translation. Thirdly, its applicability during pregnancy is limited. Originally designed for postpartum populations, the EPDS has certain constraints when used in pregnancy [105,106,107]. Common physiological discomforts during gestation (e.g., fatigue, sleep changes, appetite fluctuations) overlap considerably with depressive symptoms, making it difficult for the scale to effectively distinguish whether such symptoms originate from physiological changes or psychological pathology. This may reduce the specificity of depression assessment during pregnancy.

Beyond these psychometric limitations, screening alone does not improve outcomes unless it is linked to a clear referral and follow-up pathway. Without such integration, screening may increase detection without reducing morbidity.

### 4.2. Emerging Screening Tools and Models

To address the limitations of conventional screening tools, several innovative screening and assessment strategies have emerged in recent years, aiming to enhance the precision and efficiency of perinatal depression identification. These advancements are primarily manifested in the following three aspects. First, the optimization and innovation of screening tools have led to the development for specific populations [99,108,109,110]. For instance, the modified Patient Health Questionnaire for pregnancy (PHQ-9-P) adjusts items that overlap with common physiological experiences during gestation, such as fatigue and sleep changes [111,112]. This refinement effectively reduces interference from somatic symptoms, thereby significantly improving the specificity of depression screening in pregnant individuals. Second, the digital integration of screening processes has become a significant trend in implementation [113,114,115,116,117]. Embedding standardized questionnaires into obstetric electronic health record systems or dedicated mobile health applications enables the automation of the screening workflow. This approach not only alleviates the workload of healthcare providers but also transcends temporal and spatial constraints, substantially improving screening coverage and ensuring continuity in follow-up. Finally, the development and application of clinical prediction models represent a paradigm shift from singular metric reliance toward multidimensional risk assessment [118,119,120,121]. To address the limitation of questionnaire scores being influenced by transient emotional states, advanced models incorporating multisource data have been developed. These models employ statistical methodologies, including machine learning, to integrate diverse risk factors spanning obstetric complications, psychiatric comorbidities, and psychosocial stressors within a unified multivariate predictive framework. Through the synthesis of these heterogeneous data sources, such models facilitate enhanced risk stratification and more precise quantification of individual susceptibility, thereby establishing an evidence-based foundation for implementing early, targeted preventive interventions.

### 4.3. Emerging Biomarkers: Seeking Objective Diagnostic Evidence

Biomarker research in PND seeks to identify candidate markers and emerging auxiliary tools that may eventually support case identification and diagnosis. Nevertheless, most of the markers discussed below remain investigational and require further validation prior to clinical application.

Firstly, endocrine and neurotrophic indicators play a significant role. Evidence suggests that metabolic changes in neuroactive steroids are critical in the pathogenesis of PND. The sharp postpartum decline in ALLO levels constitutes an important pathophysiological basis [40,43]. Concurrently, reduced serum levels of brain-derived neurotrophic factor (BDNF) show a significant correlation with the severity of depressive symptoms, indicating its potential as a marker reflecting impaired neuroplasticity [82,83,84].

Second, some clinical observations show that elevated levels of pro-inflammatory cytokines, such as C-reactive protein (CRP) and interleukin-6 (IL-6), in the peripheral blood of affected individuals. These findings not only provide empirical support for the neuroinflammation hypothesis but also suggest that inflammatory markers could serve as references for subtyping the disorder or evaluating treatment response. Sominsky et al. [122] reported that compared to pregnant women with normal pre-pregnancy weight, those with pre-pregnancy obesity (BMI ≥ 30) exhibited significantly elevated levels of pro-inflammatory cytokines, such as IL-6 and tumor necrosis factor-α (TNF-α) in peripheral blood during early and mid-pregnancy. Mediation analysis revealed that increased levels of IL-6 and TNF-α significantly mediated the association between pre-pregnancy obesity and more severe antenatal depressive symptoms, indicating that a substantial portion of the depression risk attributable to obesity is mechanistically explained by the upregulation of these inflammatory markers. Furthermore, this study demonstrated that elevated levels of cytokines such as IL-6 and TNF-α can reduce the synthesis of 5-HT and brain-derived neurotrophic factor (BDNF), thereby providing additional evidence supporting their role as critical molecular players in the pathophysiology of PND.

Third, placental mechanisms offer another promising avenue. Sha et al. [123] revealed significant alterations in the enzymatic profile of the kynurenine pathway in the placentas of women with antenatal depression. Specifically, there was an upregulation of enzymes that shift the pathway toward neurotoxicity—leading to increased production of neurotoxic metabolites such as quinolinic acid (QUIN)—coupled with a downregulation of those promoting neuroprotection. This imbalance concurrently depletes the substrate for 5-HT synthesis. Together, these mechanisms drive the pathogenesis of PND. These findings indicated that the expression levels of enzymes like IDO1 in the placenta, or the ratio of kynurenine to tryptophan along with neurotoxic metabolite levels in peripheral blood, hold promise as future objective biomarkers for predicting or diagnosing PND. Furthermore, this study suggests that modulating the balance of the placental kynurenine pathway may represent a novel therapeutic strategy for preventing or treating PND.

Additionally, the role of the gut–brain axis is increasingly recognized. Xu et al. [124] reported that compared to healthy perinatal women, pregnant individuals with depression or anxiety exhibit a significant reduction in the α-diversity of their gut microbiota. β-diversity analysis further confirms distinct overall microbial community structures between the clinical and control groups. These differences are characterized by a marked decrease in beneficial bacterial genera, such as the short-chain fatty acid-producing Faecalibacterium and certain members of the Lachnospiraceae family, alongside a relative enrichment of inflammation-associated taxa like Escherichia-Shigella. Researchers hypothesize that this microbial shift may contribute to a systemic low-grade inflammatory state by reducing beneficial metabolites (e.g., butyrate) and increasing circulating pro-inflammatory agents such as lipopolysaccharide (LPS), thereby aligning with the neuroinflammation hypothesis of perinatal depression. These specific microbial signatures hold promise as minimally invasive biomarkers for predicting the risk of perinatal mood disorders.

Finally, extracellular vesicles, particularly exosomes, have garnered attention due to their ability to cross the blood-brain barrier and carry biological information from their cells of origin, including neurons. Specific microRNAs (e.g., miR-132, miR-124) detected in peripheral blood exosomes exhibit expression profiles associated with PND [125]. These molecules show promise as novel, minimally invasive biomarkers that may indirectly reflect central nervous system status, offering a new window into disease identification.

In summary, integrative approaches combining endocrine, inflammatory, placental, microbial, and vesicular markers are advancing our understanding of PND pathogenesis and paving the way for improved diagnostic and therapeutic strategies.

### 4.4. Application of Digital Technology: Passive Monitoring and Objective Early Warning

Digital technology is driving a paradigm shift in the monitoring and management of PND, enabling continuous and passive assessment of disease risk while overcoming the limitations of traditional screening methods that rely on discrete, time-point evaluations. Currently, its core applications are primarily manifested in two complementary directions. Firstly, artificial intelligence-based voice analysis technology identifies subtle patterns associated with depressive states by capturing multi-dimensional acoustic features from speech signals of pregnant or postpartum individuals. This non-invasive method provides objective quantification of dimensions such as verbal fluency and emotional expressivity, thereby offering a novel digital biomarker to assist in PND identification, with promising potential for clinical scalability [126,127,128,129]. Secondly, wearable devices such as smartwatches enable the long-term dynamic monitoring of heart rate variability (HRV), a core indicator of autonomic nervous system function. As a key parameter reflecting the balance between sympathetic and parasympathetic activity, HRV reduction typically signifies elevated physiological stress burden and compromised emotional regulation capacity, which is strongly associated with PND risk [130,131,132,133]. Through continuous tracking of HRV trends, these systems can automatically detect aberrant patterns, thereby establishing a foundation for early and objective risk stratification. The integrated application of digital phenotyping technologies signifies a transition in PND management from intermittent screening towards comprehensive, individualized risk monitoring, laying the technical groundwork for a proactive health intervention system [134].

In a culturally informed approach, Wang and colleagues [135] developed the “The Baby Coming You Ready” (BCYR) care model, which centers on digital, holistic, and strengths-based assessment. By exploring various explainable artificial intelligence (XAI) techniques, they constructed a culturally sensitive and strengths-oriented predictive model to assess perinatal psychological distress in Indigenous mothers. This study demonstrates the potential of XAI-based models in predicting psychological distress among Indigenous mothers, with the capacity to provide clear and human-interpretable explanations of how key factors interact and collectively influence outcomes. Despite the promise of digital tools, several limitations remain. Data privacy, algorithmic bias, and false positives are ongoing concerns. Digital exclusion may disproportionately affect low-income and rural populations. Moreover, most of these tools still lack external validation in diverse, real-world settings.

Overall, current approaches to screening and diagnosing PND are undergoing a paradigm shift, moving from reliance on subjective reports toward the integration of multimodal information. An ideal future framework may involve initial large-scale screening and continuous monitoring of high-risk populations using clinical prediction models and digital technologies, followed by precise assessment combining standardized scales and promising biomarkers. Such a multimodal strategy would significantly enhance the accuracy of early PND detection, creating critical opportunities for personalized intervention.

## 5. The Evolving Landscape of PND Treatment: From Traditional Protocols to Novel Interventions

The clinical management of PND has undergone considerable diversification in recent years. Therapeutic approaches have expanded beyond conventional pharmacotherapy and psychotherapy to incorporate novel targeted agents, neuromodulation techniques, and individualized precision interventions. Critical to treatment formulation is the comprehensive balancing of efficacy, safety (with particular attention to potential impacts on the fetus or infant) and accessibility to achieve an optimized clinical outcome.

### 5.1. Stepped-Care Framework

A stepwise model based on symptom severity is recommended: Mild symptoms: psychoeducation, monitoring, lifestyle interventions, peer support. Mild-to-moderate PND: CBT, IPT, guided digital therapy, collaborative care. Moderate-to-severe PND: psychotherapy plus pharmacotherapy, risk-benefit counseling. Severe, suicidal, psychotic, or treatment-resistant cases: urgent psychiatric care, specialty referral, neuromodulation, rapid-acting treatments (brexanolone, zuranolone).

### 5.2. Pharmacotherapy

#### 5.2.1. Risk–Benefit Considerations for Traditional Anti-Depressants

As summarized in Table 2, there are multiple clinical approaches to perinatal depression. SSRIs and serotonin-norepinephrine reuptake inhibitors (SNRIs) represent the most commonly utilized pharmacological interventions for perinatal depression [136]. Among these, sertraline is recommended as a first-line treatment in numerous clinical guidelines, based on its relatively favorable safety profile during pregnancy and lactation. However, late-pregnancy exposure to SSRIs has been associated with neonatal adaptation syndrome, which may present as transient tachypnea, neuromuscular symptoms, and irritability [137,138]. Meanwhile, the use of SNRIs (such as venlafaxine) remains subject to debate regarding a potential association with an increased risk of postpartum hemorrhage [139].

#### 5.2.2. Breakthrough Targeted Therapies

Brexanolone and Zuranolone, representing neuroactive steroids, signify the advent of a new era in targeted therapy for perinatal depression. Brexanolone, the first medication specifically approved by the U.S. FDA for postpartum depression, is an intravenous formulation of allopregnanolone. It acts as a positive allosteric modulator of the GABA-A receptor, rapidly counteracting the sharp postpartum decline in neuroactive steroid levels and producing significant improvement in depressive symptoms within days. The introduction of Brexanolone constitutes a major breakthrough, shifting the treatment paradigm from conventional neurotransmitter modulation to targeted intervention [140,141]. Besides, Zuranolone, an oral analogue of allopregnanolone, has demonstrated in Phase III clinical trials the ability to achieve rapid onset of action during a 14-day treatment course while sustaining therapeutic effects. Its oral administration offers a more convenient treatment option, positioning it as a promising new approach for the rapid relief of postpartum depression symptoms [142,143]. Zuranolone received FDA approval in 2023 as the first oral medication for postpartum depression in adults. However, real-world use is limited by high cost, the need for sedation monitoring (particularly with brexanolone under a Risk Evaluation and Mitigation Strategy [REMS] program), limited breastfeeding safety data (largely based on pharmacokinetic projections), and a lack of long-term outcome data beyond the acute treatment period.

#### 5.2.3. Cautious Exploration of Hormone Therapy

Estrogen supplementation therapy is derived from the “estrogen withdrawal” hypothesis, which posits that the rapid decline in estrogen levels during the perinatal period is a significant trigger for depressive episodes. However, the clinical application of this therapy remains subject to considerable controversy. Its therapeutic efficacy has yielded inconsistent conclusions across studies, lacking consistent high-level evidence for support. More critically, both pregnancy and the puerperium are inherently high-risk periods for thrombogenesis, and the administration of exogenous estrogen further elevates the risk of thromboembolic events [37,144]. Consequently, this intervention has not been incorporated into standard treatment guidelines for perinatal depression and is reserved only as an exploratory option within rigorously selected individual cases.

### 5.3. Advances in Non-Pharmacological Interventions

#### 5.3.1. Innovations in Psychotherapy

Internet-based Cognitive Behavioral Therapy (iCBT) [145,146,147] and group Interpersonal Therapy (IPT) [148,149,150,151] have significantly improved the accessibility of interventions for perinatal depression through innovations in service-delivery models. iCBT utilizes digital platforms to provide standardized, structured psychological interventions that overcome geographical and temporal barriers. In contrast, group IPT employs an organized therapeutic group format to efficiently leverage limited specialist resources, enabling simultaneous support for multiple patients. Particularly in resource-limited settings, these models offer efficient and scalable evidence-based psychological interventions, substantially expanding the coverage of perinatal mental health services.

Several studies have evaluated the efficacy of these models. For instance, Danaher et al. [152] evaluated the efficacy of the e-health program “MomMoodBooster2” for PND management. Results demonstrated that compared to the conventional care group, the e-health intervention group exhibited more significant improvements in depression severity and stress levels, particularly when integrated with universal depression screening and referral protocols, suggesting the e-health program holds substantial potential for implementation. In a comprehensive meta-analysis, Li et al. [153] evaluated the short- and long-term effects of cognitive behavioral therapy (CBT) on PND. The results demonstrated that CBT, both as a monotherapy or integrated with other approaches, was effective in reducing depressive symptoms. Significant effects were observed in the short term (SMD −0.69, 95% CI: −0.83 to −0.55) and were sustained in the long term (SMD −0.59, 95% CI: −0.75 to −0.42). While not all trials have shown preventive effects, Nishi et al. [145] found no preventive effect of iCBT on the onset of perinatal Major Depressive Episodes (MDE), they suggested that iCBT may still confer benefits in preventing perinatal depression among pregnant individuals with subthreshold depressive symptoms. Similarly, a meta-analysis conducted by Cuijpers et al. [154] on psychological treatments for PND demonstrated that psychological interventions are potentially effective in treating PND, with treatment effects lasting for at least 6–12 months. Additionally, these interventions may exert beneficial influences on social support, anxiety, functional impairment, parenting stress, and marital stress. The overall effect size was g = 0.67 [95% CI 0.45–0.89; number needed to treat = 4.39], with high heterogeneity (I^2^ = 80%; 95% CI 75–85).

Task-sharing and implementation strategies further enhance the scalability of PND interventions. Singla et al. [155] clearly demonstrated that treatment delivered by non-specialist practitioners via telephone was non-inferior to that provided by professional specialists in reducing depressive symptoms, indicating comparable core efficacy between the two delivery approaches. This task-sharing telemedicine model demonstrated high acceptability and engagement, confirming its feasibility and patient adoption in real-world settings. This study provides rigorous scientific evidence that delegating psychotherapy tasks to trained non-specialists through telephone delivery constitutes an effective, feasible, and highly promising strategy to address the substantial treatment gap in perinatal depression at scale. In a clinical trial (NCT05353491), Rahman and colleagues [156] developed a technology-assisted a peer-delivered version of the Thinking Healthy Programme (THP-TAP). The study revealed that among 846/980 (86.3%) participants assessed at 3 months postpartum, the remission rate difference was 8.91%, with a lower limit of the one-sided 97.5% confidence interval of 4.25%, which exceeded the prespecified noninferiority margin of −10% (*p* for noninferiority < 0.0001). The findings clearly demonstrate that combining trained peers with lived experience and technology-assisted delivery creates a novel intervention for perinatal depression that is noninferior to specialist-delivered therapy in efficacy, while potentially offering greater accessibility, relatability, and cost-effectiveness. Faro and colleagues [157] investigated whether the strategy of Implementation Facilitation can successfully integrate an evidence-based mental health intervention—the “Mothers and Babies” curriculum—into established maternal and infant home visiting programs in the United States. The study focuses on evaluating the effectiveness of Implementation Facilitation, which, distinct from the mental health intervention itself, functions as an “engine” or “catalyst” to promote the successful adoption of the curriculum. This research addresses a critical public health challenge in real-world settings: how to systematically integrate scientifically validated interventions into preexisting, high-workload community service systems.

Collectively, these advances underscore a shift toward more accessible, scalable, and integrative models of PND care. By combining digital tools, task-sharing, peer support, and implementation science, healthcare systems can expand the delivery of effective psychological interventions to broader populations, including those in underserved and high-risk settings. Notably, a meta-analysis of randomized trials by Miguel et al. [158] reached a clear and important conclusion: in randomized trials of psychotherapy for depression, the treatment effects (effect sizes) derived from patient self-reports are systematically and significantly larger than those based on clinician ratings. The authors strongly recommend that future clinical trials should include and report outcomes from both self-report measures (e.g., BDI, PHQ-9) and clinician-rated instruments (e.g., HAMD, MADRS) to provide more comprehensive and reliable evidence of treatment efficacy. This finding underscores that optimal clinical decision-making should integrate patient self-reports with clinician assessments, treating them as complementary rather than interchangeable sources of information.

Among these approaches, traditional CBT and IPT have the strongest efficacy evidence but limited scalability. ICBT and group IPT offer better scalability, albeit with modestly lower effect sizes. Peer-delivered and task-sharing models show comparable efficacy in resource-limited settings and may improve equity, although fidelity monitoring remains a challenge.

#### 5.3.2. Therapeutic Applications of Neuromodulation

Repetitive transcranial magnetic stimulation (rTMS) [159,160,161] and transcranial direct current stimulation (tDCS) [162,163] are non-invasive neuromodulation techniques. The strength of evidence for both modalities remains moderate, derived mostly from small or open-label studies. A common rTMS protocol involves 10–20 sessions delivered over 2–4 weeks, targeting the left dorsolateral prefrontal cortex. These techniques offer alternative options for patients with medication intolerance or inadequate response and are particularly advantageous for lactating individuals due to the absence of systemic drug exposure. However, limitations include high cost, limited accessibility, and the requirement for multiple sessions. Neuromodulation is not a routine alternative for all medication-intolerant patients; rather, it is best reserved for selected moderate-to-severe cases in specialized settings.

Esketamine has shown preliminary evidence for preventing postpartum depression in one meta-analysis; however, it remains an emerging intervention and requires perinatal safety data before clinical use.

#### 5.3.3. Lifestyle and Nutrition as a Preventive Strategy

Evidence from large-scale prospective cohort studies indicates that regular physical activity and adherence to a Mediterranean dietary pattern are associated with a significantly reduced risk of developing perinatal depression. The underlying mechanisms may involve the modulation of inflammatory responses, enhancement of neuroplasticity, and stabilization of hypothalamic-pituitary-adrenal axis function.

In terms of nutritional interventions, supplementation with eicosapentaenoic acid (EPA) and docosahexaenoic acid (DHA) demonstrates adjunct therapeutic value, particularly for individuals with low baseline serum omega-3 fatty acid levels. In a meta-analysis employing a random-effects model, Tsai et al. [164] reported that vitamin D supplementation yielded small to moderate effect sizes (SMD: 0.52; 95% CI: 0.84 to 0.20) in ameliorating PND. Therefore, it is speculated that daily intake of 1800–3500 IU vitamin D may confer some degree of therapeutic benefit for perinatal depression.

Neurobiological evidence further substantiates these findings. Jafarabady et al. [165] reported that women with PND exhibit significantly lower peripheral blood levels of BDNF compared to healthy perinatal controls. A meta-analysis demonstrated significantly reduced BDNF levels in individuals with antenatal depression (SMD: –0.31; 95% CI: –0.48 to –0.13; *p* = 0.0008; I^2^ = 71%) as well as in those with postpartum depression (SMD: –0.61; 95% CI: –0.99 to –0.22; *p* = 0.0002; I^2^ = 77%). These findings provide a neurobiological basis for the therapeutic efficacy of interventions known to elevate BDNF levels—such as regular physical exercise, certain antidepressant medications, and transcranial magnetic stimulation—in the management of perinatal depression. Furthermore, this evidence positions BDNF as a promising auxiliary objective biomarker and suggests that pharmacological agents directly targeting the BDNF system represent a viable future research direction.

Lifestyle factors such as sleep and body weight also play critical roles. Yang et al. [166] indicated that, compared to the reference group sleeping 8–9 h per day, women reporting either less than 5 h or more than 10 h of daily sleep exhibited significantly elevated risks across all outcome measures. Specifically, in the shortest sleep duration group, each additional hour of sleep was associated with a 0.79-fold reduction in the risk of neonatal low birth weight (95% CI: 0.67–0.93); conversely, in the longest sleep group, each additional hour increased the risk by 1.40-fold (95% CI: 1.06–1.84). These results suggest that abnormal sleep duration—whether insufficient or excessive—elevates associated risks, underscoring the importance of maintaining healthy sleep patterns to mitigate the risk of PND.

Similarly, Ventriglio et al. [167] confirmed a U-shaped relationship: both low BMI (underweight) and high BMI (overweight/obesity) were associated with increased risks of adverse pregnancy outcomes. Notably, the risks were particularly pronounced among women with obesity, who demonstrated significantly higher probabilities of developing gestational hypertension/preeclampsia, gestational diabetes, requiring cesarean delivery, and delivering macrosomic infants. More critically, both underweight and overweight/obese women exhibited elevated risks of perinatal depression compared to those with normal BMI. These findings highlight the necessity for enhanced screening for perinatal depression and the provision of early psychological support for women at both extremes of the BMI spectrum.

In summary, integrative approaches addressing nutrition, physical activity, sleep, and metabolic health provide a multidimensional strategy for the prevention and adjunct treatment of perinatal depression, supported by growing epidemiological and neurobiological evidence.

### 5.4. Toward Precision Medicine in Perinatal Depression

The following approaches remain emerging and speculative; none are currently validated for the routine clinical management of PND. Pharmacogenomics-guided personalized prescribing represents a key strategy for enhancing treatment response rates in perinatal depression [168,169,170]. By analyzing specific genetic polymorphisms in patients, it becomes possible to predict individual sensitivity to different antidepressants and assess the risk of adverse drug reactions. This approach enables precision medication selection, ultimately optimizing therapeutic outcomes. Moreover, Interventions targeting the microbiota–gut–brain axis offer a novel perspective for the management of perinatal depression. Research indicates that specific probiotic strains and prebiotics can exert positive effects on mood regulation through multiple mechanisms, including modulation of gut microbial composition, enhancement of intestinal barrier function, and reduction of pro-inflammatory mediators [171,172,173,174]. These microecological interventions are characterized by their high safety profile and ease of implementation, indicating considerable potential for clinical application. Pritschet and colleagues [175] noted that current approaches seeking “average” neurobiological markers and providing “standard” therapeutic regimens yield variable efficacy across individuals, with a substantial proportion of patients showing limited treatment response. To address this, the authors developed an advanced neuroimaging methodology—Precision Functional Mapping (PFM)—which involves repeated, longitudinal functional MRI scans of the same individual to delineate their unique and fine-grained “functional connectome.” By applying PFM during preconception or early pregnancy, it becomes possible to identify person-specific risk biomarkers and predict individual responses to different treatments, such as specific SSRIs, cognitive behavioral therapy, or transcranial magnetic stimulation. This work advocates a paradigm shift from “trial-and-error” interventions based on population averages toward precisely tailored interventions grounded in the functional circuitry of the individual brain.

In summary, the treatment of perinatal depression has entered a new era characterized by multimodal and personalized approaches. The future direction involves integrating rapidly acting targeted medications, highly accessible psychotherapeutic interventions, non-invasive neuromodulation techniques, and biomarker-informed preventive strategies to provide tailored therapeutic solutions for diverse patient needs.

## 6. Discussion

Several interventions for PND are already supported by high-quality evidence and are clinically actionable. These include universal screening with the EPDS, evidence-based psychotherapies (cognitive-behavioral therapy and interpersonal therapy), selective serotonin reuptake inhibitors (particularly sertraline), and collaborative care models that integrate mental health services into obstetric settings.

In contrast, a number of promising approaches remain investigational and are not yet ready for routine care. Biomarkers (e.g., brain-derived neurotrophic factor, inflammatory cytokines, microRNAs), digital phenotyping (e.g., voice analysis, wearables), microbiome-targeted interventions, precision functional mapping, and esketamine all require further validation prior to clinical adoption.

Major implementation barriers persist, including stigma, a global shortage of mental health specialists, fragmented care systems, a lack of culturally adapted screening and treatment tools, and limited access to novel treatments such as brexanolone and zuranolone due to their high cost and infrastructure requirements.

## 7. Current Challenges

Despite significant advances in the research of PND, its effective prevention, diagnosis, and treatment continue to face a series of complex challenges. These challenges span cultural, social, clinical, and scientific dimensions, requiring equally diverse and multifaceted response strategies.

### 7.1. Stigmatization and Underrepresentation

In many non-Western societies, mental health issues are heavily stigmatized, often associated with personal weakness, family shame, or supernatural causes. This stigma leads to deliberate concealment of symptoms, refusal to seek professional help, and even denial of the problem itself [3,176,177,178]. Future efforts must focus on cultural adaptation: developing culturally sensitive screening tools and leveraging community leaders and traditional support systems for mental health outreach to lower the threshold for seeking help [179].

Perinatal depression affects the entire family system, not only the mother. Studies indicate that approximately 10% of men experience depression during their partner’s perinatal period, which is associated with poorer couple communication, reduced parenting quality, and adverse child developmental outcomes. A family-systems approach is therefore needed, including: (1) integrating fathers into perinatal healthcare systems with male-specific screening tools; (2) investigating the impact of paternal depression on child development; and (3) designing couple- or family-based psychological interventions [180,181,182].

### 7.2. Resource Constraints and Clinical Decision Dilemmas

The severe worldwide shortage of specialized mental health professionals constitutes a critical bottleneck hindering the prevention and treatment of PND [183,184,185]. An effective strategy to address this challenge is to expand the group of non-specialist health workers who have received standardized training. Evidence has demonstrated that peer support programs and interventions simplified according to the mhGAP guidelines offer significant advantages in terms of acceptability and cost-effectiveness, and should be prioritized for scaling up in resource-limited settings.

Another issue is clinical decision for lactating patients requiring pharmacological treatment demands exceptional caution [186,187]. According to pharmacokinetic data, priority should be given to drugs with relatively low infant doses that are transferred to breast milk. In addition, it is essential to clearly communicate to patients and their families that the negative impact of untreated maternal depression on mother-infant interaction and early child development typically far outweighs the potential risks associated with antidepressant exposure via breast milk. This understanding is crucial for promoting treatment adherence. The final plan should be jointly developed by both doctors and patients on a fully informed basis. Shared decision-making and collaborative obstetric-psychiatric care are essential when treating lactating patients. Infant monitoring and the risk of untreated maternal depression must be carefully weighed. Protocols should be established prior to initiating antidepressants in breastfeeding mothers.

### 7.3. Translational Bottlenecks

PND research faces several inherent methodological limitations. Firstly, a critical lack of long-term follow-up data exists, as the vast majority of studies conclude at one year postpartum, failing to track children’s long-term neurodevelopmental outcomes. This gap impedes the evaluation of interventions’ lasting effects [188,189,190]. Secondly, substantial placebo effects in randomized controlled trials, particularly for psychological interventions, complicate the accurate assessment of true treatment efficacy, necessitating more rigorous control for non-specific factors. Most importantly, the field grapples with significant heterogeneity; PND likely represents not a single disease entity but a syndrome comprising subtypes with distinct underlying biological mechanisms [191,192]. This heterogeneity directly explains the wide variability in treatment response and underscores the urgent need for research aimed at defining these subtypes.

Moreover, the translation of basic research findings into clinical practice also encounters some hurdles. On one hand, existing animal models are difficult to fully simulate the complex hormonal fluctuations, social and psychological factors, and subjective emotional experiences during the human perinatal period. On the other hand, the pathway for validating and implementing biomarkers is protracted. Despite the identification of numerous potential biomarkers, their translation into clinically usable tools requires overcoming significant challenges, including standardizing detection assays, establishing clinical cut-off values, and conducting validation in large-scale prospective cohorts.

To advance biomarker research, future studies should incorporate the following: prospective cohort designs, preregistration of biomarker panels, standardized assays, external validation in independent populations, and specification of clinically meaningful outcome thresholds.

## 8. Prospect and Conclusions

In summary, addressing the outlined challenges, the future trajectory of PND research and care requires strategic focus on several critical fronts. Firstly, a primary imperative involves the systematic implementation of integrated care models [193,194]. Embedding mental health services within standard maternity care pathways to establish seamless screening, referral, and treatment processes is fundamental for improving both access and continuity of care. Secondly, it is essential to deepen the precision medicine research paradigm. Utilizing big data and artificial intelligence to integrate clinical information, genetic data, neuroimaging, and biomarker profiles will enable the definition of PND subtypes, thereby providing a scientific basis for targeted interventions and personalized treatment. Thirdly, a paradigm shift toward preemptive intervention is warranted. Prioritizing community-embedded, universal prevention initiatives before disease onset offers substantially greater long-term value compared to post-symptomatic treatment approaches [195,196,197]. Finally, strengthening interdisciplinary collaboration is imperative. A concerted effort integrating expertise from obstetrics, psychiatry, pediatrics, psychology, and public health is required to address this clinical treatment challenge.

In conclusion, addressing PND is a public health issue that demands systematic solutions. Providing genuinely effective support for affected populations requires breaking through biomedical research bottlenecks, addressing sociocultural barriers, reducing global resource disparities, and respecting patients’ participation in decision-making. Clinically, integrated care pathways should link routine screening to timely diagnostic assessment, stepped treatment according to symptom severity, emergency referral protocols for suicidality or psychosis, and longitudinal mother–infant or family follow-up. Such pathways are essential to reducing the morbidity and mortality associated with perinatal depression.

## Figures and Tables

**Table 1:** Summary of pathophysiological mechanisms in perinatal depression.

Mechanism	Key Evidence Type	Clinical Relevance	Limitations	Translational Status
Neuroendocrine (HPA axis, 5-HT, DA, NE)	Human cohort studies, biomarker assays	Supports use of SSRIs and neuroactive steroids	Correlation vs. causation; individual variability	Clinically actionable
Neuroinflammation	Cytokine levels (IL-6, TNF-α), animal models	Potential anti-inflammatory targets	Mostly observational; causal evidence lacking	Experimental
Microbiota–gut–brain axis	Human microbiome studies	Probiotic/prebiotic interventions	Small sample sizes; confounders	Exploratory
Genetic (5-HTTLPR, BDNF)	Candidate gene studies	Personalized risk assessment	Inconsistent replication	Investigational
Epigenetic (DNA methylation, miRNA)	Placental and blood studies	Early biomarkers	Validation needed	Emerging

**Table 2:** Current clinical progress in some cases of perinatal depression. NCT: National Clinical Trial.

NCT Number	Status	Conditions	Interventions
NCT06074250	Phase 2 Phase 3	Perinatal depression Perinatal anxiety	Dietary supplement: fish oil, Dietary supplement: probiotics, Behavioral: prebiotics, Behavioral: diet quality
NCT04998721	Phase 2	Depression, Postpartum efficacy, Self anxiety	Behavioral: promoting first relationships-brief Behavioral: perinatal collaborative care
NCT04685148	Phase 1 Phase 2	Major depressive disorder Postpartum depression	Drug: transdermal patch estradiol Drug: transdermal patch placebo
NCT05552053	Phase 2 Phase 3	Depression, Postpartum Perinatal depression Anxiety in pregnancy Stress, Psychological	Device: MWSH plus candlelit care Device: MWSH

## Data Availability

This review article does not report original research data. All referenced studies are publicly available as cited in the reference list.
